# Temporomandibular Joint Involvement in Juvenile Idiopathic Arthritis: The Results from a Retrospective Cohort Tertial Center Study

**DOI:** 10.3390/life13051164

**Published:** 2023-05-11

**Authors:** Artem K. Artamonov, Maria A. Kaneva, Natalia A. Gordeeva, Lubov S. Sorokina, Mikhail M. Kostik

**Affiliations:** 1 Hospital Pediatry, Saint-Petersburg State Pediatric Medical University, 194100 Saint-Petersburg, Russia; 2 Pediatric Rheumatology, Saint-Petersburg Children’s Hospital #2, n.a. Saint Mary Magdalene, 199053 Saint-Petersburg, Russia; 3 Laboratory of Autoimmune and Autoinflammatory Diseases, Almazov National Medical Research Centre, 197341 Saint-Petersburg, Russia

**Keywords:** juvenile idiopathic arthritis, temporomandibular joint, TMJ, TMJ arthritis, corticosteroids

## Abstract

Our study aimed to evaluate the clinical and laboratory features of juvenile idiopathic arthritis (JIA) children with temporomandibular joint (TMJ) arthritis. In the retrospective cohort study, we analyzed data of 753 patients with JIA aged 2–17 years, depending on TMJ arthritis or not. TMJ arthritis can to be diagnosed in the presence of at least two of the following clinical signs of inflammation: pain in TMJ, jaw opening limitation, jaw opening deviation, and micrognathia. We compared clinical, laboratory, and treatment features in JIA patients depending on the involvement of TMJ. TMJ arthritis was detected in 43 (5.7%) of our patients and associated with a longer course of the disease, polyarticular JIA category, treatment with systemic corticosteroids, and longer achievement of the remission and involvement of cervical spine, hip, and shoulder. Active joints >8 (OR = 14.9, *p* = 0.0000001), delayed remission >7 years (OR = 3.1; *p* = 0.0004), delayed hip involvement (OR = 4.6; *p* = 0.041), hip osteoarthritis (OR = 4.0; *p* = 0.014), cervical spine arthritis (OR = 10.3, *p* = 0.000001), and corticosteroid treatment (OR = 2.3, *p* = 0.0007) were associated with TMJ involvement. Patients with TMJ arthritis require more biologics (OR = 3.2, *p* = 0.0006, HR = 2.4, *p* = 0.005) and have decreased probability of remission achievement (*p* = 0.014). Consequently, TMJ arthritis was associated with a severe disease course. Early biologic treatment and corticosteroid avoidance might decrease TMJ involvement.

## 1. Introduction

Juvenile idiopathic arthritis (JIA) is the most frequent chronic inflammatory joint disease of childhood, with onset before the 16th birthday, presenting with swelling, pain, and functional disturbances of the joints with unknown etiology [1,2]. JIA encompasses different categories, according to the International League of Associations for Rheumatology (ILAR) classification [1,2]. JIA has no specific confirmatory test and requires the exclusion of other joint diseases [1]. It consists of systemic, persistent, and extended oligoarthritis, rheumatoid-factor positive and negative polyarthritis, enthesitis-related arthritis, psoriatic arthritis, and undifferentiated arthritis [1,2]. The involvement of specific joints may be associated with poor prognosis and outcomes. According to the recommendations of the American College of Rheumatology, the involvement of the hip, cervical spine, and wrist may require using biologics as a first-line treatment with or without methotrexate [3]. The involvement of temporomandibular joints (TMJ) is also suspected as a marker of severe disease course, but its diagnosis is difficult [4,5,6].

According to a recent meta-analysis, TMJ involvement is the second most common musculoskeletal disorder that causes pain and disability, with 31% of adults/elderly and 11% of children/adolescents from the general population affected, and oral diseases still a continuous major public health burden worldwide [7,8].

The clinical symptoms of TMJ arthritis are scarce and include orofacial symptoms—pain during the chewing, orofacial dysfunction, and dentofacial deformity—realized in jaw asymmetry and/or hypoplasia, malocclusion, jaw deviation during the opening of the mouth [8,9,10]. Contrast-enhanced MRI is the gold standard for the diagnostics of TMJ arthritis. Early (acute) findings include joint effusion, bone marrow edema, synovial thickening, and increased joint enhancement. Chronic dentofacial and orofacial changes are related to mandibular condyle deformity (flattening), mandibular hypoplasia, bone erosions, disk abnormalities, and subluxation [11]. It is necessary to discriminate the active TMJ arthritis and its damage and consequences, which require different management [7]. The broad use of MRI shows the prevalence of radiological findings above the clinical in TMJ arthritis. There is a gap between clinical and radiological (magnetic resonance imaging) signs of TMJ arthritis [12]. The incidence of TMJ arthritis differs from study to study, with a maximum of 96% and clinical-radiological discrepancy observed [9,12]. It is still unclear whether all patients require radiological findings to be treated or not.

Treatment approaches include NSAIDs, intraarticular TMJ injections, and non-biologic and biologic DMARDS [13]. Additionally, non-drug treatment conservative approaches are affecting pain control and functional improvement [14]. The future treatment perspectives might be related to cell technologies [15].

Several studies note that the distinct features of JIA might be associated with TMJ arthritis, but the data are contradictory [5,16]. Despite the plenty of studies about TMJ arthritis, the JIA features in patients with TMJ involvement have been scarce.

Our study aimed to evaluate the clinical and laboratory features of JIA children with TMJ arthritis.

## 2. Methods

Study design and patient selection: We extracted information from the patient’s case report forms (*n* = 753) aged 2–17 years in the retrospective single-center study (2006–2016).

Diagnosis of JIA was established according to ILAR criteria [1]. Inclusion criteria: (i) all categories of JIA [1]; (ii) Minimal two observations during at least two years periods in our center required. TMJ arthritis was diagnosed if the patient has two or more of the following clinical signs: pain in TMJ, jaw opening limitation, jaw opening deviation, micrognathia, and other orofacial deformities, related to JIA involvement. Patients were divided into two categories depending on the presence of TMJ arthritis.

Data collection: in each patient, we evaluated: (i) demographic characteristics: onset age, gender, JIA category, hip involvement, and delayed hip involvement (means the absence of hip involvement in the first six months since the onset of JIA); (ii) laboratory activity hemoglobin, white blood cells count, platelets, erythrocyte sedimentation rate (ESR), and C-reactive protein levels; (iii) treatment: treatment with corticosteroids, and their cumulative doses, the route of administration, and treatment with biologic and non-biologic disease-modifying antirheumatic drugs (DMARD)s; and (iv) the disease outcomes: achievement of inactive disease and the developing of a significant flare (means the flare, followed by changing of the current treatment).

Statistical analysis: Sample size was not calculated initially. Statistical analysis was performed with the software STATISTICA, version 10.0 (StatSoft Inc., Tulsa, OK, USA). All continuous variables were checked by the Kolmogorov–Smirnov test, with no normal distribution identified. The quantitative variables were median (Me) and percentiles (25%, 75%) for continuous variables and absolute frequencies and percentages for categorical variables. Pearson’s χ2 test or Fisher’s exact test in the expected frequencies <5 was used to compare the categorical variables. Two quantitative variables were compared using the Mann–Whitney test. The ability of each variable to discriminate patients with TMJ arthritis from patients without it was evaluated with sensitivity and specificity analysis, AUC-ROC (area under the receiver operating characteristic curve) with 95% confidence interval (CI), calculating odds ratio (OR) for the detection the best cut-offs of continuous variables. The higher values of OR of variables interfere with the better discriminatory ability. We used the “best” threshold for our data’s ROC curve analysis. Survival analysis in each group, with JIA outcomes (treatment with biologics, achievement of the remission) as the event of interest, was conducted through the Kaplan–Meier method. The log-rank test compared survival curves. Factors significantly associated with the time of JIA outcomes were then tested in a Cox proportional hazards regression model, calculating the Hazard-ratio (HR) with a 95% confidence interval (CI). *p*-value < 0.05 was considered statistically significant.

## 3. Results

### 3.1. Characteristics of JIA Patients Depending on the Presence of TMJ Arthritis

Of 753 JIA patients, TMJ arthritis was detected in 43 patients (5.7%). TMJ arthritis was associated with a longer course of the disease (5.5 years), polyarticular and systemic JIA categories, treatment with systemic corticosteroids, both orally and high-dose pulse therapy, and longer achievement of remission. TMJ arthritis was more common in patients with cervical spine, hip, and shoulder arthritis. There was a higher incidence of TMJ arthritis among patients with hip osteoarthritis and especially those who had delayed hip involvement. TMJ arthritis was rarely found among patients with the oligoarticular JIA category and who had uveitis. JIA patients with TMJ arthritis frequently received non-biologic DMARDs. Detailed characteristics of patients are in Table 1.

### 3.2. Factors Associated with TMJ Arthritis

The predictors of TMJ arthritis were selected based on the results of a single-factor analysis. The data were analyzed for sensitivity and specificity with odds ratio calculation. The predictors with the high clinical meaning were the long course of the disease, a large number of active joints, cervical spine, arthritis of the upper extremities, and hip osteoarthritis, including delayed onset. The predictors associated with TMJ arthritis are presented in Table 2.

### 3.3. TMJ Arthritis as a Predictor of Poor JIA Outcomes

**Figure 1:**
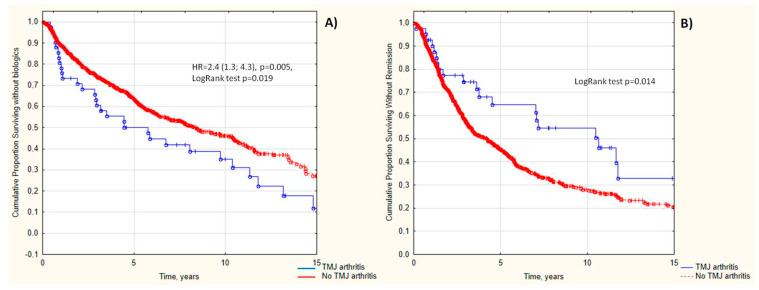
Cumulative probability of being without biologics (**A**) and being without remission (**B**) in JIA patients depending on the TMJ arthritis.

TMJ arthritis was associated with a higher probability of biologic administration (Log Rank test *p* = 0.014, HR = 2.4 (95 CI: 1.3; 4.3), *p* = 0.005) and decreased probability of achievement of remission (Log Rank test *p* = 0.014). Data are in Figure 1A,B.

## 4. Discussion

In our large single-center retrospective cohort, the predictors of TMJ arthritis were evaluated. A longer course of the disease, non-achievement of remission, polyarticular and systemic JIA categories, involvement of specific joints (cervical spine, hip, and shoulder arthritis), and systemic corticosteroid treatment were the main predictors of TMJ arthritis.

### 4.1. The Prevalence of TMJ Arthritis in JIA Patients

The prevalence of TMJ arthritis ranged from 10% to 96% [12,13] and depended on the years of the study, duration of JIA, and methods of TMJ arthritis detection—clinically, radiologically, or both [14]. Several studies did not show the involvement of TMJ among JIA patients, which is quite unusual [17,18].

Different studies showed different predictors of TMJ arthritis. In the study of Abramowicz et al., the frequency of TMJ arthritis was 10%. The authors found the following predictors: female sex, white, absence of rheumatoid factor, ANA and HLA-B27 negativity, RF-negative polyarticular JIA category, and involvement of specific joints [19]. In the study conducted by Alqanatish JT et al., the frequency of TMJ involvement was reported in 16.2% of the JIA population, 45% in polyarthritis, 20% in oligoarticular, and 15% in both systemic and enthesitis-related arthritis. Females with polyarticular JIA category and ANA-positivity were the risk factors of TMJ arthritis, while HLA B27 protected against TMJ arthritis [20].

In our study, the polyarticular course and more than eight active joints at the JIA onset were associated with TMJ arthritis. In the study of Cannizzaro E. et al., the highest prevalence of TMJ arthritis was observed in the extended oligoarthritis, and the enthesitis-related category was the least involved JIA category [21].

Patients with TMJ arthritis had a more severe disease course. The treatment with biologics (as a marker of a severe JIA course) were reported in 100% [20], 40% [21], and 24% [19]. In our study, the frequency of biologics in patients with TMJ arthritis was high (70.8%), similar to the Alqanatish JT study [20], and agrees with recent recommendations [13].

### 4.2. TMJ Arthritis-Associated Outcomes

It is necessary to distinguish active TMJ arthritis from TMJ dysfunction as a sequel of arthritis. The primary TMJ arthritis outcomes are orofacial dysfunction and dentofacial deformities. The involvement of TMJ was observed in 30.6% of patients with JIA after 18 years. The dentofacial deformities associated with TMJ arthritis, including orofacial symptoms (23.5%) and dentofacial dysfunction (52%), were seen in 20.6% of the total cohort. Age less than 9 years, female gender, and ANA-positivity were associated with TMJ involvement [22]. Orofacial symptoms and dysfunctions were typical in JIA patients (38%), especially who suffered from JIA for more than five years (53%) [4]. At least one orofacial symptom had 33% of young adults with JIA (*n* = 245) with cone-beam computed tomography (CBCT). TMJ pain, morning stiffness, and limitation on chewing were the main complaints in JIA patients. Lower mean maximal incisal opening and TMJ pain on palpation were specific for JIA patients compared to controls.

The main radiological features revealed by the CBCT were condylar deformation and/or erosions in 61% of patients, and the majority of them (70%) had bilateral involvement. Orofacial dysfunction or biological treatment were the risk factors of condylar deformities. The lowest risk of condylar deformities had patients with enthesitis-related arthritis. In our study, we observed similar risk factors, such as longer JIA duration, more involved joints, and biologic treatment typical for TMJ involvement. In our study, the association between hip osteoarthritis and delayed hip involvement (mentioned possible hip avascular necrosis) and TMJ arthritis was observed. Both conditions had similar predictors (systemic corticosteroid treatment, long JIA course, and longer non-achievement of the remission), so we can suppose similar pathogenic changes in the hip proximal epiphysis and mandibular condylar [23,24]. Patients with JIA and TMJ involvement may have active arthritis (synovial effusion and synovial hyperplasia), but some patients may have TMJ osteoarthritis as a consequence of preceding inflammation. In some patients, TMJ osteoarthritis may be found primarily without clinically evident preceding inflammation, similar to the JIA patients with primary hip osteoarthritis. Prolonged silent involvement without a clinically evident inflammatory stage, manifested by TMJ dysfunction and deformation of the mandibular condylar, suggests avascular necrosis as a leading pathogenetic factor. Interestingly, both the head of the femur and the head of the mandible have similar anatomy (a spherical joint bearing a large weight load); theoretically, they may have similarities in pathogenic changes. The management of TMJ arthritis and its consequences requires a multidisciplinary team approach with pediatric and adult rheumatologists, radiologists, orthodontists, oral and maxillofacial surgeons, orofacial pain specialists, and pediatric dentistry specialists [25].

The study had many limitations. The retrospective study design allowed the extraction of only the available clinical information from the case report histories, which might be insufficient for the comprehensive analysis. We understand that the silent involvement of TMJ might be a reason for the missing information on TMJ arthritis, and TMJ dysfunction. We are not sure that attending physicians input all information about orofacial symptoms and dentofacial deformities which might influence the study results. The absence of comprehensive dental examination and wide routine usage of TMJ of CT or MRI, which required general anesthesia, make the true frequency of the TMJ involvement unrecognized. Patients’ inclusion in the study required an observation period of fewer than five years, which might hide the actual incidence of TMJ involvement.

## 5. Conclusions

The possible targets preventing TMJ arthritis are related to routing monitoring of TMJ arthritis (contrast-enhanced MRI) in risk groups, early and prompt control of inflammation in JIA with early biologic administration in the cases of TMJ involvement, and avoidance of systemic corticosteroids. A multidisciplinary team approach is strongly recommended for the treatment of TMJ arthritis and its outcomes. Further investigations to clarify the pathogenesis of TMJ arthritis and its outcomes are required.

## Figures and Tables

**Table 1:** Characteristics of JIA patients depend on TMJ arthritis.

Parameters	TMJ Arthritis		*p*-Value
	Yes, (*n* = 48)	No (*n* = 710)	
Demography			
JIA onset age, years, Me (25%; 75%)	6.1 (2.8; 11.0)	6.0 (3.0; 10.4)	0.775
Sex, females, *n* (%)	30 (69.8)	427 (60.1)	0.209
JIA duration, years, Me (25%; 75%)	5.5 (2.7; 11.7)	4.2 (1.9; 7.4)	0.058
Uveitis, *n* (%)	1/32 (3.1)	115/488 (23.6)	0.007
JIA categories, *n* (%)			0.0006
Oligoarthritis	1 (2.3)	203 (28.6)	
Polyarthritis	25 (58.1)	240 (33.8)	
Psoriatic arthritis	2 (4.7)	38 (5.4)	
Enthesitis-related arthritis	9 (20.9)	177 (24.9)	
Systemic arthritis	6 (14.0)	52 (7.3)	
Joint involvement			
Active joints, Me (25%; 75%)	17 (10; 42)	6.0 (3.0; 11.0)	0.0001
Cervical spine, *n* (%)	24 (50)	77 (10.9)	0.000001
Shoulder, *n* (%)	12 (25.0)	44 (6.2)	0.0000001
Sterno-clavicular, *n* (%)	4 (8.3)	8 (1.1)	0.00003
Elbow, *n* (%)	17 (35.4)	103/707 (14.6%)	0.00002
Wrist, *n* (%)	23 (47.9)	181/710 (25.5)	0.00006
Metacarpophalangeal, *n* (%)	22 (45.8)	142/710 (20.0)	0.000002
Proximal interphalangeal, *n* (%)	24/43 (50.0)	168/710 (23.7)	0.000003
Distal interphalangeal, *n* (%)	11 (22.9)	59 (8.3)	0.0002
Hip, *n* (%)	13 (27.1)	140 (19.7)	0.096
Hip osteoarthritis, *n* (%)	8/13 (61.5)	40/140 (28.6)	0.014
Delay hip involvement, *n* (%)	8/10 (80.0)	58/125 (46.4)	0.041
Sacroiliac, *n* (%)	7 (14.6)	64 (9.0)	0.114
Knee, *n* (%)	36 (75.0)	499 (70.4)	0.061
Ankle, *n* (%)	28 (58.3)	295 (41.6)	0.002
Subtalar, *n* (%)	6 (12.5)	56 (7.9)	0.160
Tarsus, *n* (%)	5 (10.4)	38 (5.4)	0.085
Metatarsophalangeal, *n* (%)	15 (31.3)	83 (11.7)	0.00001
Foot interphalangeal, *n* (%)	15 (31.3)	79 (11.1)	0.000005
Laboratory data			
Hemoglobin, g/L, Me (25%; 75%)	120 (111; 128)	125 (116; 133)	0.020
White blood cells, 10^9^/L, Me (25%; 75%)	7.4 (5.9; 11.4)	7.1 (5.8; 9.2)	0.164
Platelets, 10^9^/L, Me (25%; 75%)	332 (285; 428)	311 (255;386)	0.161
Erythrocyte sedimentation rate, mm/h, Me (25%; 75%)	12 (3; 25)	7 (3; 20)	0.077
C-reactive protein, mg/L, Me (25%; 75%)	4.1 (0.0; 12.7)	1.3 (0.2; 7.4)	0.187
Rheumatoid factor, *n* (%)	1/25 (4.0)	21/381 (5.5)	0.808
ANA-positivity, *n* (%)	8/28 (28.6)	204/432 (47.2)	0.136
HLA B27, *n* (%)	4/17 (23.5)	96/291 (33.0)	0.694
Treatment			
NSAIDs, *n* (%)	42 (87.5)	613 (86.3)	0.705
Corticosteroids, *n* (%)	19 (39.6)	135 (19.0)	0.0007
Methylprednisolone pulse therapy, *n* (%)	15 (31.2)	122 (17.3)	0.032
Intra-articular corticosteroids, *n* (%)	15 (31.2)	301 (42.4)	0.116
Any corticosteroids, *n* (%)	32 (66.7)	416 (58.6)	0.252
Corticosteroid cumulative dose, mg	2050 (1000; 5000)	2850 (1000; 5000)	0.802
Non-biologic DMARD, *n* (%)	42 (87.5)	585 (82.4)	0.314
Biologic DMARD, *n* (%)	34 (70.8)	320 (45.1)	0.0006
Time before biologic, years	3.9 (1.0; 9.7)	4.2 (1.9; 7.6)	0.912
Outcomes			
Remission, *n* (%)	22 (45.8)	155 (22.0)	0.0004
Time before remission, years, Me (25%; 75%)	4.2 (1.7; 10.8)	3.1 (1.5; 6.3)	0.042
JIA flares, *n* (%)	1 (2.1)	137 (19.3)	0.005

Abbreviations: ANA—antinuclear antibodies, DMARD—disease-modifying anti-rheumatic drugs, HLA—human leukocyte antigen, JIA—juvenile idiopathic arthritis, NSAID—non-steroid anti-inflammatory drugs.

**Table 2:** Sensitivity, specificity, and odds ratio analysis of factors associated with TMJ arthritis in JIA patients.

Predictors	*Se*	*Sp*	OR (95% CI)	*p*-Value
Active joints >8	88.4	66.2	14.9 (5.8; 38.3)	0.0000001
Time before remission >7 years	46.3	78.0	3.1 (1.6; 5.8)	0.0004
JIA duration >8 years	42.9	78.6	2.8 (1.5; 5.2)	0.001
No uveitis	96.9	23.6	9.6 (1.3; 70.8)	0.007
Joint involvement				
Cervical spine arthritis	55.8	89.2	10.3 (5.4; 19.8)	0.000001
Sterno-clavicular arthritis	9.3	98.9	9.0 (2.6; 31.2)	0.00003
Hip arthritis	30.2	80.3	1.8 (0.9; 3.5)	0.096
Delayed hip involvement	53.6	80.0	4.6 (0.9; 22.6)	0.041
Hip osteoarthritis	61.5	71.4	4.0 (1.2; 13.0)	0.014
Shoulder arthritis	27.9	93.8	5.9 (2.8; 12.2)	0.0000001
Elbow arthritis	39.5	85.4	3.8 (2.1; 7,3)	0.00002
Wrist arthritis	53.5	74.5	3.4 (1.8; 6.3)	0.00006
Metacarpophalangeal joints arthritis	51.2	80.0	4.2 (2.2; 7.8)	0.000002
Proximal interphalangeal joints arthritis	55.8	76.3	4.1 (2.2; 7.6)	0.000003
Distal interphalangeal joints arthritis	25.6	91.7	3.8 (1.8; 7.9)	0.0002
Ankle arthritis	65.1	58.5	2.6 (1.4; 5.0)	0.002
Metatarsophalangeal joints arthritis	34.9	88.3	4.1 (2.1; 7.9)	0.00001
Interphalangeal foot joints arthritis	34.9	88.9	4.1 (2.1; 7.9)	0.000005
Oral corticosteroids	40.5	81.0	2.3 (1.2; 4.4)	0.0007
Corticosteroids pulse therapy	30.2	82.7	2.2 (1.1; 4.1)	0.032
Cyclosporine	30.8	89.0	3.6 (1.5; 8.8)	0.003
Biologic therapy	72.1	54.9	3.2 (1.6; 6.2)	0.0006
Non-achievement of remission	65.4	51.2	2.0 (1.1; 3.7)	0.028
No following JIA flares	19.3	97.7	10.1 (1.4; 73.7)	0.005

Abbreviations: JIA—juvenile idiopathic arthritis.

## Data Availability

The datasets generated during and/or analyzed during the current study are available from the corresponding author upon reasonable request.
